# Synergistic Enhancement of Cancer Therapy Using HDAC Inhibitors: Opportunity for Clinical Trials

**DOI:** 10.3389/fgene.2020.578011

**Published:** 2020-09-11

**Authors:** Lourdes Hontecillas-Prieto, Rocío Flores-Campos, Andrew Silver, Enrique de Álava, Nabil Hajji, Daniel J. García-Domínguez

**Affiliations:** ^1^Institute of Biomedicine of Seville, Hospital Universitario Virgen del Rocío/CSIC/Universidad de Sevilla/CIBERONC, Seville, Spain; ^2^Centre for Genomics and Child Health, Blizard Institute, Barts and The London School of Medicine and Dentistry, Queen Mary University of London, London, United Kingdom; ^3^Department of Normal and Pathological Cytology and Histology, School of Medicine, University of Seville, Seville, Spain; ^4^Division of Brain Sciences, Imperial College London, London, United Kingdom

**Keywords:** histone deacetylases, HDAC inhibitors (HDACis), combination treatment, cancer, clinical trials, preclinical studies

## Abstract

Chemotherapy is one of the most established and effective treatments for almost all types of cancer. However, the elevated toxicity due to the non-tumor-associated effects, development of secondary malignancies, infertility, radiation-induced fibrosis and resistance to treatment limit the effectiveness and safety of treatment. In addition, these multiple factors significantly impact quality of life. Over the last decades, our increased understanding of cancer epigenetics has led to new therapeutic approaches and the promise of improved patient outcomes. Epigenetic alterations are commonly found in cancer, especially the increased expression and activity of histone deacetylases (HDACs). Dysregulation of HDACs are critical to the development and progression of the majority of tumors. Hence, HDACs inhibitors (HDACis) were developed and now represent a very promising treatment strategy. The use of HDACis as monotherapy has shown very positive pre-clinical results, but clinical trials have had only limited success. However, combinatorial regimens with other cancer drugs have shown synergistic effects both in pre-clinical and clinical studies. At the same time, these combinations have enhanced the efficacy, reduced the toxicity and tumor resistance to therapy. In this review, we will examine examples of HDACis used in combination with other cancer drugs and highlight the synergistic effects observed in recent preclinical and clinical studies.

## Introduction

The role of epigenetics was first described as an essential mechanism for normal cell function. Later, epigenetic disruptions were found to promote malignant cellular transformation leading to cancer (Choi et al., 2001; Ashraf et al., 2006; Adams et al., 2010). Traditionally, cancer is considered a multistep process in which transformational events are mainly associated with genetic changes in oncogenes and tumor suppressor genes (Hanahan and Weinberg, 2011). We now know that cancer initiation and progression involves substantial epigenetic abnormalities along with other genetic alterations (Baylin and Jones, 2011; Sandoval and Esteller, 2012). Changes in the mechanisms of DNA methylation, histone modifications or small non-coding microRNAs (miRNA) are considered now as hallmarks of cancer. Understanding the epigenetic landscape of tumors constitutes a promising research area both in terms of understanding the molecular mechanism involved in tumor development and the identification of novel targets for new and combinational therapies.

Unlike genetic alterations, epigenetic changes in cancer are reversible. Such changes can be exploited as cancer epigenetic biomarkers for use in the development and evaluation of epigenetic drugs for cancer therapy. One of the promising epigenetic drugs is histone deacetylases inhibitors (HDACis). Currently, there are many preclinical studies and clinical trials testing the efficacy, toxicity and utility of different HDACis both as monotherapies and in combination with other therapies.

Histone deacetylases inhibitors, as single agents, have been shown to be effective in preclinical studies involving a wide range of molecular and biological responses in both hematological and solid tumors. In general, HDACis as monotherapy are well-tolerated and are not toxic to normal tissues in preclinical models. However, HDACis as monotherapy have had limited success in clinical trials showing only modest anti-tumor effects, especially in solid tumors, and have caused secondary effects (Munster et al., 2009). These disadvantages could be resolved, at least in part, by combining HDACis with other anticancer drugs. This strategy appears to substantially improve the conventional treatment effect in many cancer studies because of synergistic or additive antitumor effects. HDACis in combination enhance the therapeutic efficacy with respect to their effect as a monotherapy both in preclinical and clinical trials. Additionally, different combinations have shown fewer adverse effects especially in selective HDACis.

## Histone Deacetylases: An Overview

Histone deacetylases (HDACs) are a family of enzymes grouped into four classes in humans based on their homology to yeast HDACs analogs. The four classes are different in cellular localization, structure, mechanism of catalysis and expression patterns (Haberland et al., 2009) (Table 1). Class I HDACs include HDAC1, HDAC2, HDAC3, and HDAC8 enzymes located in the nucleus. Class II HDACs include HDAC4, HDAC5, HDAC6, HDAC7, HDAC9 and HDAC10 enzymes, which are located both in the nucleus and cytoplasm. Class II HDACs comprises HDACs 4, 5, 6, 7, 9, and 10 and are subdivided into Class IIA (HDAC4, HDAC5, HDAC7, and HDAC9) and Class IIB (HDAC6 and HDAC10) (Haberland et al., 2009). Class III, also called Sirtuins, include seven members of the Sirtuin HDACs from Sirtuins 1 to 7 that are located in nucleus, cytoplasm and mitochondria. Class IV is represented by HDAC11 (Haberland et al., 2009). HDACs class I, II and IV require Zinc-dependent catalysis as cofactors for their enzymatic activity and HDACs class III or Sirtuins required nicotinamide adenine dinucleotide (NAD)-dependent (Park and Kim, 2020).

**TABLE 1:** HDACs family members.

Class	Homology to yeast	Human HDACs	Deacetylase activity cofactors	Cellular localization
I	Rpd3	HDAC 1, HDAC 2, HDAC 3, and HDAC 8	Zinc dependent	Nucleus
II	Hda1	Class IIA: HDAC 4, HDAC 5, HDAC 7, and HDAC 9	Zinc dependent	Nucleus and cytoplasm
		Class IIB: HDAC 6 and HDAC 10		
III	Sir2	SIRT 1, SIRT 2, SIRT 3, SIRT 4, SIRT 5, SIRT 6, SIRT 7	NAD+ dependent	Nucleus, cytoplasm, and mitochondria
IV	HOS3	HDAC 11	Zinc dependent	Nucleus and cytoplasm

**FIGURE 1:**
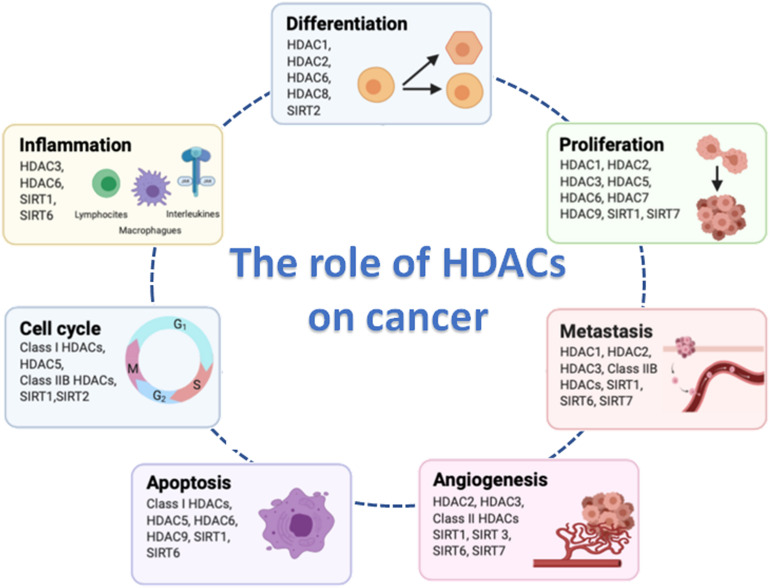
Hallmarks of cancer cell biology in which histone deacetylases (HDACs) are involved. Figure created with BioRender.com.

The main physiological function of HDACs is to maintain the steady-state level of lysine acetylation level of histone and non-histone proteins. HDACs are considered as the erasers of the acetyl group, while the histone acetyltransferases (HATs) are the writers. Balanced acetylation and deacetylation levels are controlled by the opposite activity of HDACs and HATs: HDACs are capable of removing acetyl groups from histone tail and induce a chromatin compaction; HATs induce relaxed chromatin by transferring an acetyl group from acetyl CoA to form ε-*N*-acetyl lysine. Chromatin condensation and relaxation equilibrium occurs across the whole genome, although control of the expression of a small subset of genes can occur in specific areas of the genome when a particular HDAC and HAT balance is modified. HDACs play several important roles in aspects of cancer development including tumor cell proliferation, metastasis, angiogenesis, resistance to apoptosis and alteration of the cell cycle (Figure 1). However, further studies are required to identify the specific HDACs substrates associated with these functions.

Histone deacetylases maintain steady-state acetylation levels, which allows them to play an important role in various physiological cellular functions, including differentiation, angiogenesis and metabolism (Kim et al., 2001; Fajas et al., 2002; Knutson et al., 2008). Consequently, HDACs imbalance will promote molecular changes that can influence health. Abnormal activity of HDACs has been implicated in different human diseases such as nervous system (McFarland et al., 2012), cardiovascular (Thal et al., 2012; Xu et al., 2013) and inflammatory diseases (Kim et al., 2012; Zhang et al., 2017b), and in cancer disease where their role has been explored extensively.

Alteration of HDACs activity has been associated with many types of solid tumors and hematological malignancies (Choi et al., 2001; Ashraf et al., 2006; Adams et al., 2010). The mechanisms by which HDACs contribute to cancer are diverse. In most tumors, aberrant expression of HDACs promotes oncogenic signaling by silencing tumor suppressor genes transcription or by the alteration of key target genes expression that regulate oncogenic pathways (Luo et al., 2000; Siddiqui et al., 2003; Cras et al., 2007; Godman et al., 2008; Feng et al., 2013), which are more often associated with poor outcomes in patients. For example, overexpressed of HDAC8 in neuroblastoma, cervical cancer and esophageal squamous cell carcinoma has been significantly correlated with poor prognosis in patients (Oehme et al., 2009; Vanaja et al., 2018). Many cancer cells lines, such as colon, prostate, ovarian and breast, strongly express HDAC11, which control viability and metabolic activity (Deubzer et al., 2013). Moreover, HDAC11 regulates cell cycle, apoptosis and survival in neuroblastoma cells lines (Thole et al., 2017). It has been also described that HDAC8 overexpression promotes proliferation in lung, colon, cervical cancer cells (Vannini et al., 2004) and in hepatocellular carcinoma (Wu et al., 2013). In human breast cancer cell lines overexpression of HDAC1, HDAC6, or HDAC8 contributes to increased invasion *via* increased metalloproteinase-9 (MMP-9) expression (Park et al., 2011). Furthermore, HDAC6 and HDAC8 was found to regulate cancer cell migration and invasion via α-tubulin acetylation (Wickstrom et al., 2010; Vanaja et al., 2018).

Several studies have reported a tumor suppressor role for some HDACs. For example, HDAC10 expression was found to suppresses cervical cancer cells metastasis through inhibition of matrix metalloproteinase 2 and 9 (Song et al., 2013). In contrast, low HDAC10 expression is associated with poor prognosis in lung and gastric cancer patients (Osada et al., 2004; Jin et al., 2014). Moreover, low expression of HDAC1 has been correlated with poor clinical outcomes such as metastasis and advanced stages (Chaiyawat et al., 2018) in osteosarcoma. Likewise, low HDAC3, HDAC7 and HDAC9 expressions has been associated with poor prognosis and survival in childhood acute lymphoblastic leukemia (ALL) (Moreno et al., 2010). In this study, it was shown that all the three HDACs are a promising therapeutic target for the treatment of refractory childhood ALL, although it is not clear whether an individual HDAC is more important over the others in the development of malignancy and in response to treatment.

Some studies have reported that the loss of expression and activity of certain HDACs is due somatic mutations associated with tumorigenic effects. For instance, Ropero et al. (2006), found somatic mutations of the HDAC2 gene in carcinomas with microsatellite instability caused a loss of HDAC2 protein expression which made the cells more resistance to HDACis. Additionally, Stark and Hayward described that homozygous deletions of *HDAC4* in melanomas (Stark and Hayward, 2007) and Taylor et al. (2011) identified somatic mutations in HDAC1 in 8.3% of dedifferentiated liposarcoma.

The activity of HDACs often requires the formation of a complex among with different HDACs incorporated with Sin3, NuRD (nucleosome remodeling and deacetylation), CoREST (co-repressor for element-1- silencing transcription factor), SMRT (silencing mediator of retinoid and thyroid receptors) and NCoR (nuclear receptor co-repressor) co-repressor complexes (Haberland et al., 2009). Usually in cancer, HDACs are characterized by an aberrant recruitment to these complexes. Furthermore, these co-repressor complexes can be recruited by oncogenic fusion proteins to drive tumorigenesis. For instance, in acute myeloid leukemia PML-RARα, PLZF-RARα, or AML1-ETO oncogenic fusion proteins recruit and bind to HDAC complexes increasing co-repressor activity (Gelmetti et al., 1998; Grignani et al., 1998). In Ewing sarcoma, the oncogenic fusion EWSR1-FLI1 binds to NuRD complex containing HDAC2 and HDAC3 proteins which are considered a chromatin remodeling complex that leads to the repression of gene expression (Sankar et al., 2016). The EWSR1-FLI1 fusion which regulates gene repression was reverted by HDACis leading to inhibition of histone demethylase LSD1, another NuRD complex protein. The inhibition of the formation of this complex was found to affect numerous genes including well-known target genes like *CAV1*, *NKX2.2* (Sankar et al., 2014) and subsequently enhance the anticancer efficacy.

## HDACis Treatment to Enhance the Anticancer Efficacy

The development of HDACis has improved our understanding of the molecular events that sustain the biological function of HDACs. The increased sensitivity of cancer cells to HDACis is due to the overexpression of specific HDAC isoform or group of HDACs in cancer cells. Accordingly, the altered activity or expression of the HDACs render them more sensitive to the inhibition of HDAC and subsequent induction of growth arrest, differentiation inhibition and cell death (Kim and Bae, 2011), leaving normal tissue cells unaltered. This ability to modulate gene expression via changes in acetylation status in a reversible manner place HDACs as an attractive target for the treatment of numerous cancers. Knowing which HDAC is expressed in which cancer and at what defined histopathological stage is therefore essential.

Many types of HDACis have been developed, which can be divided into different groups according to their chemical structures. HDACis can be structurally grouped into at least five classes: hydroxamates, cyclic peptides, short chain fatty (aliphatic) acids, benzamides, and sirtuin inhibitors (Kim and Bae, 2011; Eckschlager et al., 2017) (Table 2). Hydroxamates compounds are the most widely explored HDACi in preclinical and clinical studies. These molecules have been developed with a distinct chemical structure consisting of a zinc chelating group, a spacer group and an enzyme binding group that confers specificity, efficiency, and the compound’s pharmacokinetic properties (Finnin et al., 1999; Mottamal et al., 2015). Depending on its ability to inhibit HDAC classes, HDACis are classified as pan-HDAC inhibitors (pan-HDACis) that act on all HDAC classes (not including sirtuins), and selective HDAC inhibitors, that target a specific HDAC (Ceccacci and Minucci, 2016).

**TABLE 2:** Summary of chemical structures and selectivity profiles of HDAC inhibitors.

Class	HDACis	Specificity
**Hydroxamates**	Trichostatin A	I, II, and IV
	SAHA/Vorinostat	I, II, and IV
	Belinostat	I, II, and IV
	Panabinostat	I, II, and IV
	Givinostat	I, II, and IV
	Abexinostat	I, II, and IV
	Resminostat	I, II, and IV
	Quisinostat	I, II, and IV
	Riconilostat	HDAC 6
	Practinostat	I, II, and IV
	Tefinostat	I
	MPT0E028	HDAC 1, 2, and 6
	CHR-3996	I
	CUDC-101	I, II
	CUDC-907	I, II
**Cyclic peptides**	Romidepsin	I
**Short chain fatty acids**	Sodium butyrate	I, II
	Phenylbutyrate	I, II
	Valproic acid	I, II
	AR-42	I, II
	Pivanex	I, II
	AN-9	I, II
**Benzamides**	Entinostat	I, II
	Mocetinostat	I, IV
	Tacedinaline	I
	4SC-202	I
	Chidamide	HDAC 1, 2, 3, and 10
	CI944	HDAC 1 and 3
**Sirtuin inhibitors**	Nicotinamide	III
	Sirtinol	SIRT 1 and 2
	EX527	SIRT 1 and 2
	Cambinol	SIRT 1 and 2

To date, pan-HDACis have been more widely studied and used rather than selective HDACis. pan-HDACi usage started in 2006 for the treatment of different types of cancers like cutaneous T-cell lymphoma (CTCL), peripheral T-cell lymphoma or multiple myeloma (Halsall and Turner, 2016). Suberoylanilide hydroxamic acid (SAHA; Vorinostat) and Trichostatin A were first generation of HDACis. In fact, SAHA was the first approved pan-HDACis by Food and Drug Administration (FDA) for the treatment of relapsed and refractory cutaneous T-cell lymphoma (CTCL) (Gryder et al., 2012; Eckschlager et al., 2017). Following the successful results with SAHA, many other HDACis have been approved for the clinical treatment in various hematological tumors such as romidepsin or belinostat. Unfortunately, hematological tumor cells have developed drug resistance to HDACis, which promoted the regeneration and maintenance of the malignant phenotype (Wang et al., 2020). The molecular basis for drug resistance by HDACis is still unclear, although drug efflux, chromatin alteration, upregulation of oxidative stress response mechanism, defects or upregulation in apoptotic pathways have been implicated (Wang et al., 2020). Although pan-HDACis are currently approved by FDA, only limited success was achieved when used as single agents against solid tumors in clinical trials compared to the hematological malignancies. In addition, pan-HDACis cause secondary effects such as thrombocytopenia, nausea, vomiting, anorexia, and fatigue (Munster et al., 2009).

Whilst the use of pan-HDACis is relatively successful in clinical applications, the combining of HDACis with other anti-tumor drugs is now considered a major breakthrough in the treatment of both hematological and solid tumors. Recent evidence shows that combinations of HDACis with other antitumor agents increase therapeutic efficacy and decrease toxicity (Munster et al., 2009). Moreover, many studies have described their synergistic effect with different types of drugs such as topoisomerase inhibitors, PARP inhibitors, proteasome inhibitors, radiotherapy, antimetabolites, mTOR inhibitors or monoclonal antibodies amongst others. This has enabled HDACis combinational therapy to be considered as a new therapeutic strategy against solid cancers and/or drug-resistant cancers.

Combining HDACis with other anti-tumor agents may thus be a strategy to achieve their high therapeutic potential. However, side effects and toxicities from pan-HDACis still exist, which is hindering their progress in the clinic. Hence, current research efforts are focused on developing selective HDACis to reduce toxicity and thereby to overcome any adverse consequences caused by off-target effects. Although there are currently few clinical trials with selective HDACis, the results to date have been positive.

## Pan-HDACis in Combination

The use of pan-HDACis in cancer has increased considerably in the last few years. A large number of HDACis have been synthetized and tested as antitumor agents in preclinical research or in clinical trials. The following sections describe the studies performed with HDACis, focusing mainly on those synergistic combinations that have improved cancer treatment in preclinical and clinical trials over the last 5 years (Table 3).

**TABLE 3:** Summary of studies evaluating pan-HDAC inhibitors in combination with other antitumor drugs.

HDACis	Drug combination	Class of drug combination	Cancer types	Effects	Clinical status	References and trial identifier
Abexinostat	Pazopanib	Tyrosine kinase pathway inhibitors	Advanced solid tumor malignancies	Blockade VEGF pathway.	Phase I	Aggarwal et al., 2017; NCT01543763
Belinostat	Doxorubicin	Alkylant agent	Soft tissue sarcoma	Well-tolerated combination. Response rate was moderate.	Phase I/II	Vitfell-Rasmussen et al., 2016; NCT00878800
	Paclitaxel and carboplatin	Alkylant agent	Carcinoma of unknown primary site	No effects	Phase II	Hainsworth et al., 2015; NCT00873119
	Cisplatin and etoposide	Alkylant agent	Small cell lung cancer	The combination is safe.	Phase I	Balasubramaniam et al., 2018; NCT00926640
	Anti-CTLA-4 and anti-PD-1 antibodies	Immunotherapy	Hepatocellular carcinoma	Improve antitumor activity *in vivo*.	Preclinical	Llopiz et al., 2019
Butyrate	5-Azacytidine	Epigenetic drugs	Breast cancer	Blockade mammary tumorigenesis and reduction tumorosphere-forming.	Preclinical	Pathania et al., 2016
Givinostat	Doxorubicin	Alkylant agent	Different sarcomas	Cell growth reduction. Apoptosis increase. Tumor growth inhibition.	Preclinical	Di Martile et al., 2018
OKI-179	Anti-PD1 antibody	Immunotherapy	Lymphoma	HDACis sensitize lymphomas to PD1-blokage by enhance tumor immunogenicity.	Preclinical	Wang et al., 2019
Panobinostat	Bortezomib and dexamethasone	Proteasome inhibitor	Multiple myeloma	Increase in progression free survival.	Phase III	Richardson et al., 2016; NCT01023308
	Carfilzomib	Proteasome inhibitor	Multiple myeloma	Combination safe and effective.	Phase I	Kaufman et al., 2019; NCT01549431
	Radiation	Radiotherapy	Bladder cancer	Increase growth delay in tumor xenografts.	Preclinical	Groselj et al., 2018
Quisinostat	Flavopiridol	Tyrosine kinase pathway inhibitors	Cutaneous and uveal metastatic melanoma	Cell proliferation reduction. Cell cycle arrest and apoptosis inhibition. Tumor growth inhibition.	Preclinical	Heijkants et al., 2018
	Flavopiridol	Tyrosine kinase pathway inhibitors	Cutaneous melanoma	Tumor growth inhibition and regression.	Preclinical	Heijkants et al., 2018
Valproic acid (VPA)	Cisplatin	Alkylant agent	Lung cancer	Induction of apoptosis and cell cycle perturbation.	Preclinical	Gumbarewicz et al., 2016
SAHA	Cisplatin	Alkylant agent	Larynx cancer	Cell proliferation suppression. Induce cell cycle arrest and apoptosis.	Preclinical	Grabarska et al., 2017
	Cisplatin	Alkylant agent	Lung cancer	Induction of apoptosis and cell cycle perturbation	Preclinical	Gumbarewicz et al., 2016
	Doxorubicin	Alkylant agent	Lymphoma	No study results posted	Phase I/II	NCT00785798
	Rituximab-CHOP	Alkylant agent	Diffuse large b-cell lymphoma	Increased toxicity	Phase I/II	Persky et al., 2018; NCT00972478
	Bortezomib	Proteasome inhibitor	Cervical cancer	Potent anti-tumor effects and lead to tumor-specific immunity.	Preclinical	Huang et al., 2015
	Carfilzomib	Proteasome inhibitor	B-cell lymphomas	No effects or modest effects	Phase I	Holkova et al., 2016; NCT01276717
	Sapanisertib	Tyrosine kinase pathway inhibitors	Lung cancer	Oxidative stress	Preclinical	Malone et al., 2017
	Ridaforolimus	Tyrosine kinase pathway inhibitors	Renal carcinoma and other solid tumors	No molecular effects	Phase I	Zibelman et al., 2015
	AZD1775	Tyrosine kinase pathway inhibitors	Head and neck squamous cell carcinoma	WEE1 inhibition leads to mitotic disfunction and increase DNA damage and apoptosis.	Preclinical	Tanaka et al., 2017
	131I-Metaiodobenzylguanidine	Radiotherapy	Neuroblastoma	Good outcome	Phase I	DuBois et al., 2015; NCT01019850
	Chemoradiation therapy	Radiotherapy	Head and neck squamous carcinoma	Combination safe and effective.	Phase I	Teknos et al., 2019
	Radiation	Radiotherapy	Pancreatic cancer	Induction of apoptotic response.	Preclinical	Moertl et al., 2019
	HCI-2509	Epigenetic drugs	Ewing sarcoma	Cell cycle arrest. Induction of apoptosis. Migratory capacity inhibition.	Preclinical	Garcia-Dominguez et al., 2018
	Decitabine	Epigenetic drugs	Acute myeloid leukemia	Stabilization of marrow disease.	Clinical trial	Glasser et al., 2017
	Ex917	Epigenetic drugs	Rhabdomyosarcoma	Cell death induction.	Preclinical	Haydn et al., 2017
	Pembrolizumab	Immunotherapy	Non-small lung cancer	Preliminary anti-tumor activity.	Preclinical	Gray et al., 2019
	Anti-CTLA-4 and anti-PD-1 antibodies	Immunotherapy	Breast cancer	Reduction tumor growth and increased survival.	Preclinical	Terranova-Barberio et al., 2017b

### Pan-HDACis in Combination With Alkylating Agents

Alkylating agents are used in standard cancer treatments that result in elevated toxicity to normal tissues due to the high used doses. For this reason, several preclinical studies have been conducted over the past 5 years in which these anticancer agents have used lower doses combined with HDACis to reduce toxicity. Currently, preclinical studies have shown greater efficacy when cells were pretreated with HDACis prior to exposure to DNA damaging agents (Suraweera et al., 2018). Based on this treatment strategy, combination therapies with HDACis and alkylating agents as topoisomerase II inhibitors have led to higher nuclear topoisomerase II inhibition accumulation, increased DNA damage, growth inhibition and cell death (Suraweera et al., 2018). In general, the molecular mechanism of HDACis sensitizing cancer cell to DNA damaging agents includes both a mechanistic action by inducing chromatin relaxation and increased accessibility to the exposed DNA and increased binding of transcription factor to reactivate the transcription of proapoptotic genes.

Pan-HDACis reduce tumor growth by inhibiting cancer cell proliferation. For instance, SAHA, combined with cisplatin, was considered as a potential treatment for larynx cancer cells due to the synergistic effect on cell proliferation inhibition (Grabarska et al., 2017). Also, SAHA and TSA was found to synergize with cisplatin in different tumor types, including cholangiocarcinoma cells, inducing cell growth inhibition (Asgar et al., 2016). Furthermore, the effect of cisplatin combined with SAHA or VPA on different cell lines of lung cancer results in synergistic response (Gumbarewicz et al., 2016). For example, SAHA was shown to suppressed cell growth of small-cell lung cancer (SCLC) by causing cell cycle arrest, and, in combination with cisplatin, significantly reduce the expression of Notch1. This is likely beneficial for this particular cancer type where oncogenic Notch1 signaling clearly plays an important role (Wawruszak et al., 2019).

More recently, a specific class I and II HDACi ITF2357 (Givinostat) was shown to induce mitochondrial apoptosis by increasing pro-apoptotic BH3 proteins and a caspases-dependent mechanism (Di Martile et al., 2018). Givinostat combined with doxorubicin (a dual topoisomerase inhibitor I and II and DNA damage agent) has shown significant potential in improving sensitization in different preclinical models of sarcoma such as osteosarcoma, liposarcoma, synovial sarcoma, rhabdomyosarcoma, leiomyosarcoma, or fibrosarcoma (Di Martile et al., 2018).

Histone deacetylases inhibitors can act as potentiators of the cytotoxicity generated by anthracycline-type topoisomerase (topo) II inhibitors (Munster et al., 2009). Following successful preclinical studies, a number of trials have been conducted in patients. For instance, promising results were obtained by combining SAHA and doxorubicin in patients with relapsed or refractory multiple myeloma (NCT00744354) or advanced/refractory lymphoma (NCT00785798) (Suraweera et al., 2018). Another alkylating agent used in solid cancers (e.g., small cell lung cancer) is etoposide; both etoposide or cisplatin have been used in combination with belinostat (PXD101), a hydroxamic acid- type HDACi approved by the FDA, to enhance their efficacy in clinical trial phase I (NCT00926640) (Balasubramaniam et al., 2018). Belinostat is well-tolerated and its combination with conventional cancer therapies has identified no further bone marrow toxicity. This has enabled the use of belinostat in several Phase I/II clinical trials in both hematological cancers and solid tumors such as ovarian cancer, multiple myeloma, adult acute myeloid leukemia and bladder cancer.

Some clinical studies have not had the same outcome. For example, a phase I/II study in patients with advanced stage diffuse large B-cell lymphoma (NCT00972478), in which SAHA was combined with Rituximab-CHOP (treatment based in vincristine and prednisone, a DNA damage inductor and a DNA synthesis inhibitor respectively) showed increased toxicity, particularly neutropenia and sepsis (Persky et al., 2018). A phase II study in patients with carcinoma of unknown primary origin (NCT00873119) showed that the addition of belinostat to paclitaxel/carboplatin did not improve the progression-free survival (PFS) (Hainsworth et al., 2015). Furthermore, a phase I/II trial using a combination of belinostat and doxorubicin in soft tissue sarcoma (NCT00878800) did not provide clear evidence of synergy (Vitfell-Rasmussen et al., 2016). These contrary results might be explained by mechanisms of anticancer effects of HDACis not being identical, and it is also possible that in combinational therapy one drug may interfere or modulate the mode of action of the other (Santoro et al., 2016). Although HDACis in combination with DNA damaging agents have shown promising results in certain types of cancer, only very limited positive outcomes have been identified in others. This depends on the cancer type and the HDACi-drug combinatorial regimes. The understanding of the combination mechanism using relevant preclinical models should improve the selection of the most appropriate combination for specific type of cancer. Also, understanding the mechanistic reasons for unexpected trial results is necessary in order to inform improved rationales for combining HDACis with cytotoxic drugs.

### Pan-HDACis in Combination With Proteasome Inhibitors

The high proliferation of cancer cells requires an elevated protein synthesis rate; this makes them highly dependent on the ubiquitin-proteasome system and, therefore, more susceptible to proteasome inhibitors (Goldberg, 2007). Following this reasoning, preclinical and clinical studies have been performed. In fact, the combined treatment SAHA and a proteasome inhibitor, bortezomib, enhances the antitumor effects and immune properties of cervical cancer cells, both *in vitro* and *in vivo* (Huang et al., 2015). Furthermore, synergistic effect was observed when bortezomib and dexamethasone were combined with panobinostat in a Phase III (NCT01023308) trial for multiple myeloma patients. This combination worked due to the panobinostat action on different epigenetic and protein metabolism pathways reducing the resistance of tumors in these patients to the treatment of proteasome inhibitors (Richardson et al., 2016). These results paved the way for other studies such as a phase I study (NCT01549431) in patients with relapsed or refractory multiple myeloma (RRMM) which has responded positively to the panobinostat as pan-HDACi that exerts activity on class I, II and IV HDACs combined with carfilzomib, a proteasome inhibitor (Kaufman et al., 2019). In contrast, a modest response was obtained in phase I study (NCT01276717) conducted with a combination of proteasome inhibitor carfilzomib and vorinostat in patients with relapsed or refractory B-cell lymphomas (Holkova et al., 2016). While, carfilzomib with Panobinostat combination is safe and effective in RRMM patients, future trials should explore the molecular mechanism of this combination with different doses in order to optimize treatment tolerability and enhance efficacy.

### Pan-HDACis in Combination With Tyrosine Kinase Pathway Inhibitors

Tyrosine kinases are involved in multiple signaling pathways regulating a multitude of biological cell functions. Deregulation of tyrosine kinases are closely associated with cancer development and progression, driving the development of many tyrosine kinase inhibitors.

The multityrosine kinase inhibitor of vascular endothelial growth factor receptor (VEGFR), and other growth factor receptors drugs, have shown significant clinical efficacy in multiple tumor types, although different cancers will readily develop resistance against it. To reduce this resistance, a study in phase I focused on patients with advanced solid tumor malignancies, and used a combination of pazopanib with pan-HDACi abexinostat (PCI24781) (NCT01543763) which resulted in a synergistic effect (Aggarwal et al., 2017). Several mechanisms can be associated with this positive effect, although abexinostat inducing the downregulation of HIF-1α protein expression is one of the more plausible mechanisms that can directly regulate VEGF expression. In this clinical trial is was not clear which HDAC or class of HDACs are involved in regulating VEGF-driven expression in tumors (Aggarwal et al., 2017).

Preclinical studies also reported a synergistic effect between HDACis and tyrosine kinase inhibitors. Both mTOR and its pathway are relevant in cancer studies. It has been shown that mTOR inhibitors exert only cytostatic effects in some NF1-associated malignancies (Malone et al., 2017). The Vorinostat/Sapanisertib combination was identified as a promising drug combination that kills NF1-mutant nervous system malignancies as well as NF1- and KRAS-mutant lung cancers. This combination triggered irresolvable oxidative stress in NF1-mutant malignancies *in vitro* and *in vivo*, but did not kill normal cells and was not toxic to mice *in vivo* (Malone et al., 2017). Moreover, another inhibitor of mTOR pathway, ridaforolimus, has been used combined with vorinostat in a phase I study of advanced renal cell carcinoma and other solid tumors (Zibelman et al., 2015). The combination was tolerated by patients in phase II and the possible mechanism shown to involve the downregulation of various cell cycle regulators such as pAkt and HIF-1α expression. Furthermore, tyrosine kinases such as WEE1 and CDKs are implicated in many signaling pathways. WEE1 is a kinase that has been linked to G2/M arrest and drugs cytotoxic action (Mackintosh et al., 2013). A preclinical study showed a synergistic effect between vorinostat and AZD1775 (WEE1 inhibitor) *in vitro* and *in vivo* in head and neck squamous tumor cells expressing high-risk mutant p53 (Tanaka et al., 2017). The combined effect of quisinostat (pan-HDACi) and flavopiridol (CDKi) was a promising therapeutic strategy for both cutaneous and uveal metastatic melanoma (Heijkants et al., 2018). *In vitro* results showed a synergistic reduction of cell proliferation and a cell death increase in uveal melanoma cell lines. Moreover, quisinostat and flavopiridol effectively reduced tumor growth *in vivo*, in a patient-derived xenograft model of cutaneous melanoma (Heijkants et al., 2018). This study suggested further investigation of the quisinostat and flavopiridol combination in the clinic was warranted since melanoma patients generally have limited therapeutic options and both drugs are already in clinical trials.

### Pan-HDACis in Combination With Radiotherapy

Radiotherapy remains one of the most common treatments for cancer. The therapy causes double-stranded DNA breaks that induce cell death. The capacity of tumor cells to repair radiation-induced DNA damage can be decreased by HDACis, by affecting DNA damage signaling and repair pathways (NHEJ and HR) (Groselj et al., 2013). Therefore, combining HDACis and radiotherapy has gained traction in cancer clinical trials. A study in phase I in patients with relapsed or refractory neuroblastoma showed that vorinostat combined with a radiosensitizer such as 131I-Metaiodobenzylguanidine (131I-MIBG) was well-tolerated by patients (NCT01019850) (DuBois et al., 2015). Similarly, vorinostat in combination with chemoradiation therapy in phase I trial of neck squamous cell carcinoma patients was found to be safe and highly effective (Teknos et al., 2019). Preclinical studies using SAHA combined with radiation have demonstrated an increase in tumor radiosensitivity *in vitro* and *in vivo* and a subsequent induction of apoptotic response in, for example, pancreatic adenocarcinoma cancer cell lines (Moertl et al., 2019). Another pan-HDACi, panobinostat were tested in a bladder cancer xenograft model and shown to be an efficient tumor radiosensitizer with no increased toxicity (Groselj et al., 2018). In this preclinical study, HDACi induced radiosensitization effects were associated with the DNA repair MRE11-RAD50-NBS1 (MRN) complex, which is known to be regulated primarily by HDAC class I enzymes.

### Pan-HDACis in Combination With Other Epigenetic Drugs

Epigenetic mechanisms are somewhat interlinked and inter-dependent. For example, HDACs and LSD1/KDM1A play important roles in regulating this interaction and, as a consequence, have emerged as promising therapeutic targets (Ning et al., 2016). Here, a combination of different epigenetic drugs may enhance efficacy. For example, the combination of a LSD1 inhibitor (HCI-2509) and SAHA exhibited a synergistic inhibition on tumor growth and enhanced apoptosis in Ewing sarcoma cell lines and in patient-derived xenograft mouse models (Garcia-Dominguez et al., 2018). Similarly, another combination of Ex917 (LSD1 inhibitor) and SAHA reported a synergistic induction on cell death in rhabdomyosarcoma cells (Haydn et al., 2017).

An alternative approach under examination is the use of DNA methyltransferase inhibitors (DNMTis) and HDACis combinations. Both DNMTs and HDACs silence gene expression, so their inhibition can be used to enhance tumor suppressor gene expression in various malignancies. Combination of 5-azacytidine (DNMTi) and butyrate (HDACi) efficiently blocked mammary tumorigenesis and reduced the tumorosphere-forming potential of tumor-propagating cells *in vitro* by reactivating the relevant tumor suppressor genes (Pathania et al., 2016). In addition, a very small scale clinical trial with two patients for relapsed or elderly secondary myelodysplastic syndrome and acute myeloid leukemia, investigated treatment with decitabine (DNMTi) and vorinostat (HDACi). This combination therapy achieved stabilization of marrow disease, outpatient palliation, and family-reported reasonable quality of life (Glasser et al., 2017). A combination of different HDACis/DNMTis could be beneficial in high-risk multiple myeloma patients. The authors developed a score for the prediction of primary multiple myeloma cell sensitivity to HDACi/DNMTi (TSA/decitabine and vorinostat/5-azacitidine) (Bruyer et al., 2018), which could be used in follow-up studies.

### Pan-HDACis in Combination With Immunotherapy

In the last few years, immune checkpoint inhibitors (ICIs) such as anti–programmed cell death protein 1 (anti-PD-1) and anti-programmed cell death ligand 1 (anti-PD-L1) have been developed. Due to the relevance of HDAC/HAT pathways in regulating the immune system, HDACis have been considered as immunomodulatory agents (Banik et al., 2019), thus they have been combined with ICIs. Pembrolizumab (an anti-PD1 inhibitor) with vorinostat was well-tolerated in advanced/metastatic non-small cell lung cancer and anti-tumor activity was demonstrated (Gray et al., 2019). Another combination strategy is the use of antibodies, anti-PD-1 or anti-CTLA-4. The novel HDACi, OKI-179 which inhibits class I, IIb and IV HDACs, sensitized resistant lymphoma cells to anti-PD1 *in vitro* (Wang et al., 2019). The results from combined OKI-179/anti-PD1 antibody treatment in lymphomas showed a synergistic effect compared with more limited effects in monotherapy (Wang et al., 2019). Moreover, the results obtained previously with anti-CTLA-4 antibody use in patients with advanced hepatocellular carcinoma (Duffy et al., 2017) encouraged the use of a combination of anti-CTLA-4 and anti-PD-1 antibodies with the pan-HDACi, belinostat (Llopiz et al., 2019). These combinations were shown to potentiate the already known efficacy of these antibodies by reducing tumor volume and improving their immune functions in a murine hepatocellular carcinoma model (Llopiz et al., 2019). A similar study used a triple combination with vorinostat/anti-CTLA-4/anti-PD-1 in the triple-negative 4T1 breast cancer mouse mode (Terranova-Barberio et al., 2017b). The synergistic interaction of the three drugs resulted in a significant increase in survival and anti-tumor activity in established mouse breast cancer allografts (Terranova-Barberio et al., 2017b).

## Selective HDACi in Combination

The intention behind the relatively recent development of specific-HDACis is to improve efficiency and broaden the therapeutic window whilst reducing the adverse effects such as thrombopenia, neutropenia, diarrhea, nausea, vomiting, and fatigue that are associated with pan-HDAC inhibition (Munster et al., 2009; Parbin et al., 2014). This section describes the studies that have used selective HDACis over the last 5 years in both preclinical and clinical trials (Table 4).

**TABLE 4:** Summary of studies evaluating selective HDACs inhibitors in combination with other antitumor drugs.

HDACis	Drug combination	Type of drug	Cancer type	Effects	Clinical status	References and trial identifier
Chidamide (CS055)	Doxorubicin	Alkylant agent	T-cell lymphoma	DNA damage protein p-γH2AX and apoptosis proteins upregulation. Anti-apoptosis protein Bcl-2 downregulation.	Preclinical	Zhang et al., 2017a
Citarinostat (ACY-241)	Pomalidomide	Immunotherapy	Multiple myeloma	Myc and IRF4 pathways and the pro-survival factor surviving suppression.	Preclinical	North et al., 2017
Entinostat	Aldesleukin	Immunotherapy	Renal cell carcinoma	No study results posted	Phase I/II	NCT01038778
LC-0296	Cisplatin	Alkylant agent	Head and neck cancer	ROS balance alterated,	Preclinical	Alhazzazi et al., 2016
				compromising cell survival and enhancing apoptosis.		
MPT0G413	Bortezomib	Proteasome inhibitors	Multiple myeloma	Enhanced polyubiquitinated protein accumulation and apoptosis. Reduced tumor cell viability and growth.	Preclinical	Huang et al., 2019
Nexturastat A	5-Azacytidine	Epigenetic drugs	Ovarian cancer	Type I interferon response upregulated. Enhanced cytokine production, and MHC I expression on the cell surface.	Preclinical	Moufarrij et al., 2020
	Anti-PD-1 therapy	Immunotherapy	Melanoma	Decreased anti-inflammatory phenotype of macrophages and down-regulated immunosuppressive proteins in tumor cells.	Preclinical	Knox et al., 2019
Nicotinamide	Doxorubicin	Alkylant agent	Breast cancer	Cell growth and cell migration inhibition. Enhanced cell apoptosis by SIRT1/Akt pathway.	Preclinical	Wei et al., 2019
RGFP966	Anti-PD-L1 therapy	Immunotherapy	B-cell lymphomas	Modulate immune-related genes to enhance anti–PD-L1 therapy.	Preclinical	Deng et al., 2019
Ricolinostat (ACY-1215)	Bendamustine	Alkylant agent	Lymphoma	Apoptosis induction by increasing ROS. Caspase-8, -9, and 3 activation. Bcl-2 proteins family modulation.	Preclinical	Cosenza et al., 2017
	Oxaliplatin	Alkylant agent	Colorectal cancer	Downregulation of p-ERK and p-AKT and induction of cell apoptosis via activation caspase-3 and elevation of the Bak to Bcl-xL ratio.	Preclinical	Lee et al., 2018
	Bortezomib and dexamethasone	Proteasome inhibitors	Multiple myeloma	Autophagic protein degradation.	Preclinical	Vogl et al., 2017
	Carfilzomib	Proteasome inhibitors	Multiple myeloma	Inductions ER stress and enhance apoptosis.	Preclinical	Mishima et al., 2015
	Bortezomib	Proteasome inhibitors	Multiple myeloma	No study results posted	Phase I/II	NCT01323751
	Ibrutinib	Tyrosine kinase pathway inhibitors	Lymphoma	Inhibition of p-IRE1 and p-BTK. Tumor growth delay.	Preclinical	Amengual et al., 2017
	Lenalidomide and dexamethasone	Immunotherapy	Multiple myeloma	Antitumor activity.	Phase Ib	Yee et al., 2016; NCT01583283
	Anti-PD-L1 therapy	Immunotherapy	Ovarian carcinoma	Tumor progression limitation in a cytotoxic T-cell.	Preclinical	Fukumoto et al., 2019
Romidepsin	Erlotinib	Tyrosine kinase pathway inhibitors	Non-small cell lung cancer	Combination well-tolerated, with evidence of disease control and exhibits effects on relevant molecular targets.	Phase I	Gerber et al., 2015; NCT01302808
Sirtinol and AGK2	Dichloroacetate acid (DCA)	Tyrosine kinase pathway inhibitors	Lung cancer	Decreased glucose consumption and lactate production. Increased OCR and ROS generation.	Preclinical	Ma et al., 2018
Tenovin-6	Metformin	Tyrosine kinase pathway inhibitors	Lung cancer	Accumulation of p53 acetylation and induction of the apoptotic pathway.	Preclinical	Lee et al., 2019

### Selective HDACis in Combination With Alkylating Agents

Combinations of selective HDACis with different alkylating agents have shown synergistic antitumor effects. A selective HDAC6 inhibitor, ricolinostat (ACY-1215) has been combined in different tumors in preclinical studies. Ricolinostat and Bendamustine combination promoted a higher apoptosis induction than when drugs were used as single agents in lymphoma cell lines (Cosenza et al., 2017). The same effects were observed in colorectal cell lines combining ricolinostat with oxaliplatin (Lee et al., 2018). A novel selective HDACi, CS055 (Chidamide) was combined with doxorubicin in peripheral T-cell lymphoma cell lines (PTCL) in a preclinical study that identified a synergistic antitumor effect when this combination was used to treat PTCL cell lines (Zhang et al., 2017a).

Nicotinamide and LC-0296 are selective class III HDAC inhibitors or Sirtuins. The combination of nicotinamide (SIRT1 inhibitor) and doxorubicin increased the inhibition of cell proliferation and apoptosis and reduced resistance to treatment in breast cancer cells (Wei et al., 2019). A novel Sirtuin-3 inhibitor, LC-0296, was developed and its effect studied in head and neck cancer cells. This preclinical study showed how this HDACi inhibited cell survival and proliferation and promoted apoptosis both in monotherapy and in combination with cisplatin despite the resistance of this cancer cells to the alkylating agent described (Alhazzazi et al., 2016). Furthermore, the selective Sirt1 inhibitor the indole EX-527 shows a significant effect in distinct types of cancer, as monotherapy or combined to cancer drugs. However, the efficacy of this Sirt1 inhibitor requires further investigation and subsequent review (Rifai et al., 2018).

### Selective HDACis in Combination With Proteasome Inhibitors

Preclinical studies in multiple myeloma conducted prior to the last 5 years (Hideshima et al., 2005) have encouraged exploration of different combinations based on selective HDACis and proteasome inhibitors.

Ricolinostat was combined with bortezomib and dexamethasone for relapsed or refractory multiple myeloma. The selective inhibition of HDAC6 combined with this proteasome inhibitor triggered dual blockade of aggresomal and proteasomal degradation of protein, and therefore synergistic multiple myeloma cell death (Vogl et al., 2017). Similar results were obtained with the same combination *in vitro* and *in vivo*, which caused a significant delay in tumor growth and prolonged overall survival in a xenograft mice model (Amengual et al., 2015). Ricolinostat had also been combined with carfilzomib promoting synergistic anti−multiple myeloma effects, even in bortezomib−resistant cells. A decrease in tumor volume associated with apoptosis in an *in vivo* mouse xenograft was also demonstrated (Mishima et al., 2015). In addition, a novel selective-HDAC6 inhibitor, MPT0G413, was evaluated, *in vitro* and *in vivo*, in combination with bortezomib. This showed a synergistic inhibition of multiple myeloma tumor cell viability (Huang et al., 2019). All available preclinical studies have supported clinical trials in multiple myeloma patients with the ACY-1215 and bortezomib combination (NCT01323751).

### Selective HDACis in Combination With Tyrosine Kinase Pathway Inhibitors

Sirtinol and AGK2 are two selective inhibitors of SIRT2. These inhibitors were combined with dichloroacetate acid (DCA), a pyruvate dehydrogenase kinase inhibitor in a preclinical study in lung cancer cells (Ma et al., 2018). Both combinations showed a synergistic effect in proliferation inhibition and apoptosis induction *in vitro*, and a reduction of tumor volume in mice (Ma et al., 2018). In addition, the combination Sirtinol/AGK2/DCA enhanced synergistically all the effects describe previously *in vitro* and *in vivo* (Ma et al., 2018).

Tenovin-6, an inhibitor of both SIRT1 and SIRT2, in combination with metformin, an antidiabetic drug and mTOR signaling pathway inhibitor, caused cell growth inhibition and induced apoptosis in lung cancer cells (Lee et al., 2019). Additionally, a preclinical study identified the synergistic interaction of ricolinostat and ibrutinib the first-in-class BTK (Bruton’s tyrosin kinase) inhibitor in a large panel of lymphoma cell lines and in a xenograft model of lymphoma. This combination led to a marked tumor growth delay and prolonged overall survival (Amengual et al., 2017).

A Phase I study in patients with non-small cell lung cancer studied the combination of an EGFR inhibitor, erlotinib, and a selective HDACi, romidepsin (NCT01302808). This combination was based on the involvement of an HDACi to counter erlotinib resistance, although the addition of romidepsin did not appear to alter the frequency and severity of characteristic erlotinib toxicities, such as rash and diarrhea (Gerber et al., 2015).

### Selective HDACis in Combination With Other Epigenetic Drugs

Combining nexturastat A (selective HDAC6 inhibitor) with 5-azacytidine (DNMTi) has been described recently as a novel approach for ovarian cancer. Results showed a significant increase of the type I interferon response and cytokine expression in 5/6 ovarian cancer cell lines *in vitro* compared to each individual treatment. Moreover, a synergistic decrease of PD-L1 protein in ovarian cells was found, which suggested that nexturastat A/5-azacytidine increased tumor immunity. However, this combination only showed a synergistic decrease in tumor burden at week 7 *in vivo* (Moufarrij et al., 2020).

### Selective HDACis in Combination With Immunotherapy

RGFP966, a novel selective HDAC3 inhibitor, enhanced the efficacy of anti-PD-L1 therapy in the treatment of B-cell lymphomas. In fact, tumor regression in syngeneic murine B-cell lymphoma model was observed in the combinatorial therapy (Deng et al., 2019). In a similar way, a HDAC6 inhibitor (riconilostat) blocked the immune checkpoints when it was combined with anti-PD-L1 therapy in ovarian carcinoma cell lines and in *in vivo* models (Fukumoto et al., 2019).

HDAC6 inhibitors are frequently used in preclinical studies in cancer because of their immunomodulatory properties. Ricolinostat potentiated the effects of lenalidomide (immunomodulatory drug) and dexamethasone in a phase Ib in patients with multiple myeloma (NCT01583283) (Yee et al., 2016). ACY-241 (citarinostat) suppressed proliferation and viability of tumor cells derived from multiple myeloma in combination with pomalidomide. This combination also improved cytotoxicity by decreasing apoptosis and cell cycle arrest (North et al., 2017). A recent study showed that nexturastat A in combination with anti-PD-1 antibodies significantly decreased tumor growth *in vivo* compared to each agent alone when treating melanoma cells (Knox et al., 2019).

A phase I/II clinical trial (NCT01038778) has studied the combination of entinostat (a selective HDAC1 and HDAC3 inhibitor) and aldesleukin (interleukin 2) in renal cell carcinoma patients. The results showed a greater median progression-free survival without increased toxicity.

## HDACis and Multitherapy

Although most of the combinations described evaluation of two drugs, multitherapy evaluate three or more different compounds. This approach is based on the fact that tumors are heterogeneous in their mutational status and involve multiple pathways in their oncogenesis and progression. Also, three-way or more therapeutic combinations may reduce the possibility of resistance, which so often limits single drug usage, and improve treatment efficacy. SAHA was part of a combination with two antimetabolites, cladribine and Gemcitabine and an alkylating agent, busulfan. This preclinical study in lymphoma cell lines demonstrated that any increase in cytotoxicity could be attributed to stable chromatin relaxation mediated by the antimetabolites and SAHA, thereby increasing the susceptibility of genomic DNA to busulfan alkylation (Ji et al., 2016). SAHA was also used in a multitherapy approach in clinical trials. The results of SAHA in combination with radiotherapy led to further trials by adding an alkylating agent to the combination. This strategy was used in the temozolomide phase I/II studies in patients with glioblastoma (NCT00731731) (Galanis et al., 2018) or included antimetabolites such as capecitabine in patients with non-metastatic pancreatic cancer (NCT00983268) (Chan et al., 2016). Like SAHA, other HDACis have been used: belinostat has also been combined with radiation therapy and temozolomide to treat glioblastoma (NCT02137759) (Gurbani et al., 2019); and valproic acid (HDACi) combined with capecitabine and radiotherapy in colorectal cancer where a synergistic antitumor interaction *in vitro* was shown (Terranova-Barberio et al., 2017a).

The development of a drug that acts on multiple targets is a new therapeutic approach that currently is being evaluated with positive results. Romidepsin and its analogs, FK-A5 and FK-A11, have showed a dual inhibition on HDACs and PI3K resulting in stronger cytotoxic effects in a panel of human cancer cell lines (Saijo et al., 2015). CUDC-101 is a strong inhibitor against HDACs, EGFR and HER2. A Phase I Study combined CUDC-101 with chemoradiation in head and neck squamous cell carcinoma patients at biologically efficacious doses was tolerated (Galloway et al., 2015). Other studies demonstrated the antitumor activity of CUDC-101 in EGFR-overexpressing glioblastoma, anaplastic thyroid cancer (Zhang et al., 2015; Liffers et al., 2016) and in pancreatic cancer when CUDC-101 was combined with gemcitabine (Ji et al., 2018).

## Conclusion

The overexpression of HDACs in hematological and solid tumors places HDACis as a promising strategy for the treatment of cancer patients. The early success of HDACis in the treatment of cancer were due to the use of pan-HDACis in hematologic malignancies. The effectiveness of pan-HDACis for cancer treatment relies on its broad-spectrum inhibition of various HDACs. This has triggered numerous studies which have investigated the antitumor effects of these epigenetic drugs. However, toxicities and unintended effects were also observed. All of which has contributed to the development of new strategies.

**FIGURE 2:**
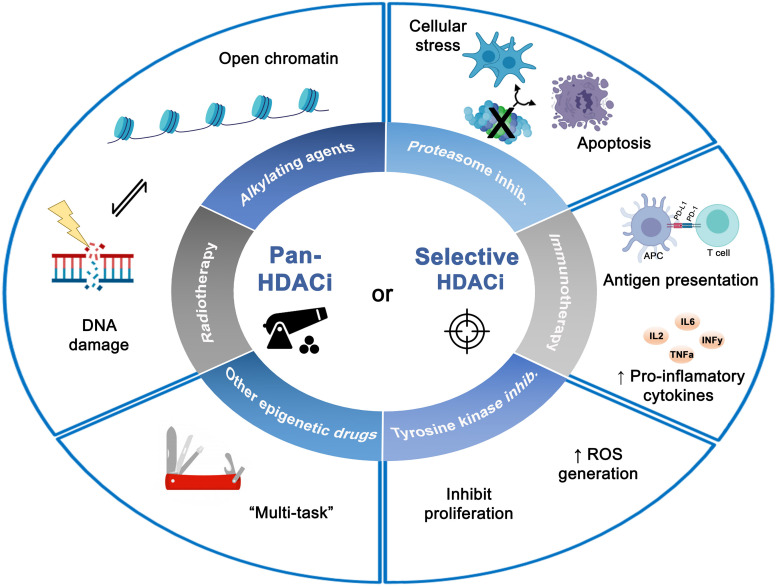
Effects of combined histone deacetylases inhibitors (pan- or selective HDACis) with different antitumor therapies on tumor cells. The diverse combinations with HDACis showed synergistic antitumor effects leading to a wide spectrum of biologic effects such as DNA damage, apoptosis induction, inhibition of proliferation and cellular stress. Figure created with BioRender.com.

The use of pan-HDACis in combination with other antitumor agents was the strategy pursued first. The diverse combinations of pan-HDACis with proteasome inhibitors, tyrosine kinase inhibitors, alkylating agents, radiotherapy, immunomodulators have revealed synergistic antitumor effects in many types of cancers both in the preclinical and clinical settings. However, such combinations often trigger various side effects such as fatigue, thrombocytopenia and gastrointestinal issues. Hence, selective HDACis have been developed with higher selectivity and specificity. The difference in increased expression and activity of specific HDAC isoform in tumors but not in normal tissues, have led to the hypothesis that selective HDACis may possess a better therapeutic index, fewer adverse effects, and better patient outcomes with cancer treatment. The current results using selective HDACis in combination with antitumor treatment have had positive results in preclinical and in early clinical studies. In addition, it has been shown that selective HDACis not only reduce the toxicity but also replicate the same effects obtained by those pan-HDACis that target the specific HDAC of selective HDACis (Figure 2).

Apart from the advantages of combined HDACis improving cancer treatment, these strategies have increased our knowledge about HDACs mechanisms and their inhibitory effect on tumors. This is particularly important in developing rationales for selecting HDACis for combination therapy.

Although preclinical studies showed that the combination of HDACis with anticancer agents might prove more effective than current therapies, the results obtained in clinical trials have not always been completely successful. This might be explained because many studies are not based on a deep understanding of the molecular events that underpin the synergistic effect of the combinations or are at present ill-defined. It also remains possible that one chemotherapy drug may interfere, or modulate the mode of action of the other (Santoro et al., 2016). A further possibility is that most preclinical research does not test different doses to select the best for use in combinations. This is essential if we are to increase the efficacy in clinical trials and reduce side effects. Importantly, we need to endeavor to understand the mechanistic reasons for reduced drug efficacy in combination trials, as investigating unexpected results will help inform the development improved rationales for selecting HDACis/cytotoxic drug combinations. Improving novel drug combinations with existing therapies need to be based on a thorough understanding of the molecular mechanism involve in cancer cell killing. Also, the study of pharmacodynamics biomarkers as indicators of drugs effects will be of significant use in evaluating the link between drugs regimen, target effects, and biological tumor response. Informative biomarkers means better evaluation of efficacy through improved treatment monitoring. Unfortunately, biomarkers remain a largely unmet clinical need and for those that are potentially available, our knowledge of their pharmacodynamics is limited.

HDACi as single agents have a limited utility in clinical and its combination with anticancer agent’s triggers adverse effects related to toxicity, safety, and efficacy. Accordingly, the use of single HDACis with multiple targets is another attractive and new alternative against solid and drug-resistant tumors, which have been gaining increasing attention. In fact, HDACis are being modified and equipped with additional biochemical activities. This might imply a potent antitumor activity with a better toxicological profile. However, it is a challenge to develop and design these multi-target HDACis.

Epigenetic therapy appears to be a promising and beneficial strategy given the successful results obtained in the treatment of several tumors with HDACis (especially in combination) and with multi-target HDACis. These attractive strategies could be useful for treating those tumors with a low rate of somatic mutations in which epigenetic plays a more central role in oncogenesis and tumor progression (e.g., rhabdoid tumor, Ewing sarcoma, or acute myeloid).

Further studies are needed to understand the mechanisms of action of HDACis in order to increase the efficacy in clinical trials and to reduced side effects. Further intensive investigations are required to provide a firm basis for the successful use of HDACis as anti-cancer agents in the clinic.

## Author Contributions

LHP and DGD conceived and wrote the manuscript, and contributed to the figure and table design. RFC wrote the manuscript and contributed to the figure and table design. AS, EDÁ, and NH wrote the manuscript. All authors revised and approved the final version for publication.

## Conflict of Interest

The authors declare that the research was conducted in the absence of any commercial or financial relationships that could be construed as a potential conflict of interest.
